# Telepsychiatry for patients with movement disorders: a feasibility and patient satisfaction study

**DOI:** 10.1186/s40734-019-0077-y

**Published:** 2019-06-06

**Authors:** Andreea L. Seritan, Melissa Heiry, Ana-Maria Iosif, Michael Dodge, Jill L. Ostrem

**Affiliations:** 1 Department of Psychiatry, University of California, 401 Parnassus Ave, Box 0984-APC, San Francisco, CA 94143 USA; 2 University of California, San Francisco Weill Institute for Neurosciences, San Francisco, USA; 3 Department of Neurology, University of California, San Francisco, San Francisco, California USA; 4 Department of Public Health Sciences, University of California, Davis, Davis, California USA; 5 University of California, San Francisco, School of Medicine, San Francisco, USA

**Keywords:** Telemedicine, Telepsychiatry, Movement disorders, Parkinson’s disease, Feasibility, Patient satisfaction

## Abstract

**Background:**

Telemedicine is a convenient health service delivery modality for patients with movement disorders, including Parkinson’s disease (PD), but is currently underutilized in the management of associated psychiatric symptoms. This study explored the feasibility of and patient satisfaction with telepsychiatry services at an academic movement disorders center.

**Methods:**

All patients seen by telepsychiatry between January and December 2017 at the UCSF Movement Disorders and Neuromodulation Center were invited to participate. Participation was voluntary. Patients received an initial survey after the first telepsychiatry visit and satisfaction surveys after each visit. Survey responses were collected online via Research Electronic Data Capture (REDCap). Frequencies were calculated for categorical variables, and means and standard deviations were generated for continuous variables.

**Results:**

Thirty-three patients (79% with PD; 72% Medicare recipients; 64% men; mean age, 61.1 ± 10.5 years; mean distance to clinic, 79.9 ± 81.3 miles) completed a total of 119 telepsychiatry and 62 in-person visits. Twenty-two initial surveys and 50 satisfaction surveys (from 21 patients) were collected. Patients were very satisfied with the care (95%), convenience (100%), comfort (95%), and overall visit (95%). Technical quality was somewhat lower rated, with 76% patients reporting they were very satisfied, while 19% were satisfied. All patients would recommend telemedicine to friends or family members.

**Conclusions:**

Telepsychiatry is a feasible option for patients with movement disorders, leading to high patient satisfaction and improved access to care. Technical aspects still need optimization. Whenever available, telepsychiatry can be considered in addition to in-person visits. Future studies with larger samples should explore its impact on patient care outcomes and caregiver burden.

## Background

Movement disorders, particularly Parkinson’s disease (PD), are often associated with psychiatric manifestations. Anxiety and depressive disorders are the most common psychiatric comorbidities, estimated to occur in at least half of PD patients, although they may be under-recognized and not addressed effectively [1–3]. Poor access to mental health resources further limits the patients’ ability to receive appropriate treatment for psychiatric comorbidities. In a recent survey of 769 PD patients, 52% reported lack of mental health services in their area, and 31% identified transportation as a barrier to mental health care [3]. Additionally, patients with movement disorders have difficulty driving long distances to attend appointments, especially to clinics located in large cities, where traffic and parking are constant challenges. PD patients may also experience unpredictable off-medication states that can be associated with dystonia, pain, wearing-off related anxiety, depression, or cognitive fluctuations. These symptoms, whether physical or psychological, make it challenging for patients to fully engage in psychiatric care.

Telemedicine offers a potential convenient alternative for this patient population. There is a growing body of literature exploring the use of telemedicine, particularly for the neurological care of PD patients. Multiple studies have shown that telemedicine is a feasible option for PD patients, leading to better motor performance and quality of life and cost savings for patients and health care systems [4–7]. Despite these encouraging initial successes, not all studies have shown improvements in patient quality of life [8, 9]. Previous authors also found high levels of satisfaction among patients and providers, concluding that telemedicine is a useful adjunct to in-person treatment and it improves access to care for patients residing in remote locations [5–7, 9].

To date, telemedicine has been used with good results for a variety of mental health conditions and age groups. A comprehensive review and meta-analysis of internet-based psychotherapy interventions for anxiety disorders, depression, posttraumatic stress disorder, alcohol use, and smoking cessation revealed moderate to large effect sizes [10]. Telepsychiatry has been shown to increase older adults’ access and satisfaction with health care services [11–13]. However, there is limited evidence regarding the use of telemedicine for the management of PD psychiatric comorbidities [14]. A small pilot study evaluated internet-administered cognitive behavioral therapy (CBT) for the treatment of depression and anxiety in nine PD patients and found a significant reduction in their depressive symptoms [15]. Other authors delivered CBT interventions by telephone to PD patients with anxiety and depression, although they did not use videoconferencing [16–19]. The Parkinson’s Active Living program was an innovative behavioral activation intervention for apathy delivered via telehealth to non-demented patients with PD [20]. This program led to significant improvements in apathy and depression, and benefits were maintained one month later [20].

The present study aimed to explore the feasibility of and patient satisfaction with telepsychiatry services offered in a busy academic movement disorders center in California. California has large rural areas, where access to mental health providers is limited. Thus, a secondary goal of our study was to increase access to psychiatric services for patients with movement disorders from a broader catchment area. To our knowledge, this is the first study to explore the use of telepsychiatry for patients with movement disorders.

## Methods

### Setting

The University of California San Francisco (UCSF) Movement Disorders and Neuromodulation Center (MDNC) is a large interprofessional center dedicated to diagnosing and treating patients with movement disorders, many of whom receive deep brain stimulation (DBS) therapy. This setting and the patient population served were described in detail elsewhere [21]. The team includes a geriatric psychiatrist. In an effort to increase patient access to mental health services, especially for those who reside in remote areas, psychiatry follow-up visits can be provided by telemedicine. Currently, the US federal health insurance Medicare does not reimburse this telemedicine model in California (medical office to patients' homes), however UCSF sponsors these services. Patients have to be located in California during the visit, have the requisite technical capability (computer with camera and speakers, tablet or phone with internet connection), and not be considered a safety risk (suicidal or violent). UCSF uses Zoom, a web-based, encrypted, HIPAA-compliant application (available at https://zoom.us/) to connect with patients in their homes. Telepsychiatry allows more frequent mental health visits. Patients typically see their neurologist approximately every three months, and the psychiatrist every four-to-six weeks. In-person appointments are coordinated so patients can see both their neurologist and psychiatrist every three months, with one-to-two interim telepsychiatry visits. The follow-up duration varies depending on patient needs (DBS evaluation and perioperative care, or regular mental health care for psychiatric conditions associated with the movement disorders), and is typically six months-to-one year.

Telepsychiatry visits are structured like regular psychiatric outpatient follow-up visits, including medication management and psychotherapy. Visits include a review of psychiatric symptoms, discussion of medication benefits and side effects, and any other stressors the patient wishes to discuss. Treatment recommendations typically include behavioral strategies and/or medication adjustments.

### Participants

All patients seen by the MDNC psychiatrist via telemedicine between January 1, 2017 and December 31, 2017 were invited to participate. Participation was entirely voluntary. The study protocol was reviewed by the UCSF Institutional Review Board and determined to not meet criteria for human subject research.

### Data collection

Participating patients or their designated caregivers received an initial survey after the first telemedicine visit, and satisfaction surveys after each visit (including the first one) by email. Emails containing the survey link were sent after the completion of each visit, with a reminder a week later. Survey responses were collected via REDCap (Research Electronic Data Capture), a secure, web-based application designed for research study data collection and storage [22].

The initial survey was developed for this study and included six demographic questions (patient age, sex, education level, marital status, employment status, and approximate distance from their residence to MDNC). This survey explored participants’ previous experience with telemedicine and opinions regarding telemedicine, as compared to in-person visits (rated on a Likert-type scale: *“Not as good”*, *“As good as”*, and *“Better than”* in-person visits). Participants were asked to rank advantages (*“I don’t have to drive and park at UCSF”*, *“It saves time”*, *“It’s more comfortable to have the visit in my home”*) and disadvantages *(“Loss of privacy”*, *“I prefer face-to-face communication with my doctor”*, and *“Technical difficulties”*) of telemedicine visits on a Likert-type scale from 1 to 3, with 1 = *Most important*, and 3 = *Least important*.

The visit satisfaction survey was adapted, with permission, from a survey used in the Remote Access to Care, Everywhere, for Parkinson Disease (RACE-PD) study [5] and assessed the level of patient satisfaction with several aspects of the visit (technical quality, care received, convenience, comfort, and overall satisfaction), rated on a 5-point Likert-type scale from 1 = *Very satisfied* to 5 = *Very dissatisfied*. Participants were also asked if they would recommend telemedicine to friends or family members and invited to comment on specific visit aspects that they liked or did not like, as well as share any additional feedback.

### Data analysis

Feasibility was assessed by the percentage of completed telemedicine visits. Descriptive statistics were used to summarize the data: frequencies (percentages) for categorical variables, and means and standard deviations (SDs) for continuous variables. Differences in characteristics between the patients who provided initial surveys and those who did not were assessed using Fisher’s exact test for categorical variables and Wilcoxon two-sample test for continuous variables (since some of these variables were skewed). For satisfaction surveys where more than one survey was available for a patient, survey variables were first summarized within patient, by calculating median values. In secondary analyses, the association between patient satisfaction scores and distance from patients’ home to MDNC was analyzed, using Spearman correlations.

## Results

Thirty-three patients (26 of whom had PD) completed a total of 119 telepsychiatry and 62 in-person visits. Of 124 scheduled telemedicine visits, there were five no-shows (96% visit completion rate), for the following reasons: missed appointments (*n* = 3), and *n* = 2 patients with severe physical and/or cognitive limitations had no family members available to help connect them to Zoom at the time of the visit. Visits lasted 30–60 min. Twenty-two patients returned initial surveys, which were completed by patients (*n* = 18) or their caregivers (*n* = 4). Of the 11 patients who did not complete initial surveys, only two received surveys and did not return them. Nine patients did not receive surveys, for the following reasons: clerical error (*n* = 1), visits took place before the IRB final determination (*n* = 3), and *n =* 5 patients were not sent surveys because they were severely ill and the team felt that completing the surveys would be too burdensome for their caregivers. These five patients’ diagnoses included: Alzheimer’s disease, multiple system atrophy, pantothenate kinase-associated neurodegeneration with generalized dystonia, psychosis, and posttraumatic stress disorder with functional movement disorder. For the patients who did not complete initial surveys, demographic and clinical data were collected by chart review.

Patient characteristics are summarized in Table 1. Patients ranged in age from 22 to 74 years old, and the distance from their homes to UCSF ranged from 6 to 400 miles. Three patients did not have movement disorders; the MDNC psychiatrist receives occasional referrals from outside the center due to the subspecialty training (geriatric psychiatry). Patients had multiple concurrent psychiatric diagnoses (range, 1 to 5; mean, 2.5 diagnoses per patient), most common being depressive disorders (79%), anxiety disorders (70%), and neurocognitive disorders (39%). Twenty (61%) patients had co-occurring anxiety and depressive disorders. There were several significant differences (*p* < 0.05) between patients who completed initial surveys and those who did not: age (non-completers were younger), education (non-completers had lower level), and number of telemedicine and total visits (non-completers had fewer visits). Twenty-four (72%) patients had Medicare as their primary health insurance.

**Table 1:** Patient demographic and clinical characteristics

	All Patients	Completed Initial Surveys		
		Yes	No	*P*-value
Variable	(*n* = 33)	(*n* = 22)	(*n* = 11)	
Age (years), *mean* (*SD*)	61.1 (10.5)	64.3 (6.9)	54.6 (13.6)	0.02
Male gender, *n* (*%*)	21 (64%)	16 (73%)	5 (45%)	0.15
Education (years), *mean* (*SD*)	17.0 (3.0)	17.7 (3.1)	15.5 (2.3)	0.04
Marital status, *n* (*%*)				1.00
Single - never married	3 (9%)	2 (9%)	1 (9%)	
Married	26 (79%)	17 (77%)	9 (82%)	
Divorced	4 (12%)	3 (14%)	1 (9%)	
Employment status, *n* (*%*)				0.50
Working full-time	6 (18%)	3 (14%)	3 (27%)	
Working part-time	1 (3%)	1 (5%)	0 (0%)	
On disability	9 (27%)	5 (23%)	4 (36%)	
Retired	16 (48%)	13 (59%)	3 (27%)	
Unemployed	1 (3%)	0 (0%)	1 (9%)	
Distance from clinic (miles), *mean* (*SD*)	79.9 (81.3)	81.9 (88.7)	75.9 (67.6)	0.98
Living in rural areas	13 (39%)	9 (41%)	4 (36%)	1.00
Neurological disease, *n* (*%*)				0.19
Parkinson’s disease	26 (79%)	19 (86%)	7 (64%)	
Other^a^	7 (21%)	3 (14%)	4 (36%)	
Surgical status, *n* (*%*)				0.31
Deep brain stimulation surgery	20 (61%)	15 (68%)	5 (45%)	
Thalamotomy	1 (3%)	1 (5%)	0 (0%)	
No surgery	12 (36%)	6 (27%)	6 (56%)	
Psychiatric diagnosis^b^, *n* (*%*)				
Depressive disorders^c^	26 (79%)	17 (77%)	9 (82%)	1.00
Anxiety disorders^d^	23 (70%)	16 (73%)	7 (64%)	0.70
Cognitive disorders^e^	13 (39%)	10 (45%)	3 (27%)	0.46
Anxiety and depressive disorders	20 (61%)	13 (59%)	7 (64%)	1.00
Total visits per patient, *mean* (*SD*)	5.5 (3.2)	6.6 (3.0)	3.1 (2.2)	0.001
In-person, *mean* (*SD*)	1.9 (1.4)	2.0 (1.2)	1.5 (1.6)	0.14
Telemedicine, *mean* (*SD*)	3.6 (2.7)	4.6 (2.8)	1.5 (0.9)	0.001

Due to rounding, percentages might not add up to 100. *P*-values from nonparametric tests: Wilcoxon for continuous variables and Fisher’s exact test for categorical variables

^a^Includes: Alzheimer’s disease; chronic pain; dystonia; essential tremor; functional movement disorder; multiple sclerosis; multiple system atrophy

^b^Subcategories total more than 100%; patients had multiple psychiatric diagnoses

^c^Includes: major depressive disorder, dysthymia, and depressive disorder due to another medical condition, unspecified, or not otherwise specified

^d^Includes: generalized anxiety disorder, panic disorder, social anxiety, and anxiety disorder due to another medical condition, unspecified, or not otherwise specified

^e^Includes: mild neurocognitive disorder and major neurocognitive disorder

Table 2 shows initial survey responses with regard to patients’ prior experience with telemedicine, perceptions of telemedicine as compared to in-person treatment, and advantages and disadvantages of telemedicine visits. Almost half of patients (45%) had previous exposure to telemedicine, either for psychiatric visits or appointments with other providers. When compared to in-person visits, 12 (55%) patients felt telemedicine would be just as good, 5 (23%) perceived it as not as good, while 5 (23%) regarded telemedicine as a better alternative. None of the five patients who thought telemedicine would be superior had had previous telemedicine experience.

**Table 2:** Initial survey responses (*n* = 22)

Survey question	*n* (%)
Previous experience with telemedicine	10 (45%)
Telemedicine compared to in-person	
Not as good	5 (23%)
As good as	12 (55%)
Better than	5 (23%)
Most important advantage of telemedicine visits^a^	
No need to drive and park at UCSF	15 (68%)
Saves time	4 (18%)
More comfortable	3 (14%)
Second most important advantage of telemedicine visits^a^	
No need to drive and park at UCSF	5 (23%)
Saves time	10 (45%)
More comfortable	7 (32%)
Third most important advantage of telemedicine visits^a^	
No need to drive and park at UCSF	2 (9%)
Saves time	8 (36%)
More comfortable	12 (56%)
Most important disadvantage of telemedicine visits^a,b^	
Prefer face-to-face communication	11 (52%)
Technical difficulties	7 (33%)
Loss of privacy	3 (14%)
Second most important disadvantage of telemedicine visits^a,b^	
Prefer face-to-face communication	7 (33%)
Technical difficulties	7 (33%)
Loss of privacy	7 (33%)
Third most important disadvantage of telemedicine visits^a,b^	
Prefer face-to-face communication	3 (14%)
Technical difficulties	7 (33%)
Loss of privacy	11 (53%)

UCSF = University of California, San Francisco

Due to rounding, percentages might not add up to 100

^a^Ranked on a Likert-type scale from 1 = *Most important* to 3 = *Least important*

^b^Missing 1 response (incomplete survey)

Patients ranked the advantages of telemedicine visits in order of importance as follows: *“I don’t have to drive and park at UCSF”*, *“It saves time”*, and *“It’s more comfortable to have the visit in my home”*. Patients completed the visits at home, in their car, or at work (for those who were still working). The main disadvantage of telemedicine visits was, *“I prefer face-to-face communication with my doctor*”, and the least important was loss of privacy (see Table 2).

Twenty-one of the 22 patients who completed initial surveys also filled out visit satisfaction surveys. A total of 50 satisfaction surveys were collected (range, 1 to 6; mean, 2.4 surveys per patient). Responses are summarized in Table 3. Almost all patients reported being very satisfied with the care received (95%), convenience (100%), comfort (95%), and overall visit (95%). Only 76% of patients stated they were very satisfied, and 19% were satisfied, with the technical quality of the connection during the visit. All respondents stated they would recommend telemedicine to friends or family members. Table 4 illustrates examples of patient and caregiver comments selected from the open-ended survey responses.

**Table 3:** Visit satisfaction survey responses (*n* = 21)^a^

Survey question	*n* (%)
How satisfied were you with the technical quality of the connection during the visit?	
Very satisfied	16 (76%)
Satisfied	4 (19%)
Neutral	1 (5%)
How satisfied were you with the care you received during the visit?	
Very satisfied	20 (95%)
Satisfied	1 (5%)
Neutral	0 (0%)
How satisfied were you with the convenience of the visit?	
Very satisfied	21 (100%)
Satisfied	0 (0%)
Neutral	0 (0%)
How satisfied were you with the comfort of the visit?	
Very satisfied	20 (95%)
Satisfied	1 (5%)
Neutral	0 (0%)
How satisfied were you with the visit overall?	
Very satisfied	20 (95%)
Satisfied	1 (5%)
Neutral	0 (0%)

^a^*n* = 21 patients provided 50 visit satisfaction surveys

Questions were rated on a 1–5 Likert-type scale, with: 1 = *Very satisfied*, 2 = *Satisfied*, 3 = *Neutral*, 4 = *Dissatisfied*, 5 = *Very dissatisfied*. For patients who provided more than one visit satisfaction survey, scores were first summarized within-person, by calculating the median satisfaction score. There were no *Dissatisfied* or *Very dissatisfied* responses for any of the questions

**Table 4:** Examples of patient and family member comments

Tell us what you liked about your telemedicine visit: *“Really happy about privacy, comfort, saving time. There is no downside.”* *“I didn’t think I’d like telehealth, but I’ve grown to like it due to convenience. I do not think telehealth should ever replace in-person visits completely.”* *“It’s good to be seen in person but when physically not able to travel telemedicine is a very good option.”* *“So happy that this is available so that I can continue my relationship with Dr. XXX.”* *“Ability to have an appointment between office visits.”* *“We were able to bring in all my husband’s caretakers including myself for a group discussion.”* *“We feel very relaxed during the visit there is no stress associated with rushing into the office through the busy traffic which is great.”* *“I felt better cared for than with office visits.”*	
Tell us what you did NOT like about your telemedicine visit: *“I really enjoy seeing my doctor in person.”* *“Set up of Zoom is not easy.”* *“Personal contact is usually better with someone with dementia; however, in this case the visit worked out fine as [it was] difficult to get patient to a visit.”* *“I expected a follow up or note describing what action I should take.”*	

The correlations between patient satisfaction scores and the distance from patients’ home to MDNC were modest, ranging from − 0.11 (for technology) and 0.26 (for care received and overall visit).

## Discussion

The present study underscores the feasibility, high patient satisfaction, and improved access to mental health care achieved by providing telepsychiatry services to patients with movement disorders. Both patients and providers easily adopted this novel treatment modality. The 96% visit completion rate illustrates the high patient adherence to telepsychiatry in a busy interprofessional clinic serving complex patients, such as the UCSF MDNC. Previous work has highlighted the feasibility of telemedicine for the neurological care of patients with PD [4–9]. However, the present study is the first to specifically explore mental health services delivery in an integrated care movement disorders clinic. Similar to previous studies of telemedicine for PD patients [5, 6], the present study revealed very high (95–100%) satisfaction levels among patients with various neurological impairments. Technical aspects were lower rated (only 76% of patients indicated being very satisfied), as also noted by other authors [4].

Our team provided psychiatric management via telemedicine to patients from a wide catchment area, many of whom would not have otherwise had access to mental health services. Poor access to mental health care providers is a universal problem, especially for older adults or those with limited mobility [5, 12]. Over two thirds (72%) of our patients had Medicare; there is a well-known shortage of Medicare providers, particularly geriatric psychiatrists, in California and nationally [23]. Furthermore, Medicare reimbursement for telemedicine services is restricted to rural areas, greatly limiting its availability [24]. In the present study, all qualifying patients, regardless of health insurance and geographic location, were offered telemedicine. As previously shown by Dorsey et al., [5] we found that telemedicine improved access to care for patients with movement disorders and other neurological conditions.

Telepsychiatry allowed close monitoring of patients with various neurological and psychiatric illnesses, including during the DBS perioperative period. Although rare, psychiatric complications associated with DBS therapy may occur, including depression, impulsivity, mania, psychosis, and suicidal ideation or behavior [21, 25]. Close psychiatric follow-up is warranted, especially for patients who are deemed to be at higher risk for psychiatric complications. About two thirds (64%) of patients in the present study had received surgical treatment for PD or essential tremor, which typically indicates more advanced neurological disease. Patients also had a high lifetime prevalence of anxiety (70%) and depressive (79%) disorders, and 61% had both anxiety and depressive disorders. Due to the high co-occurrence of these diagnostic categories and the small overall sample size, patient perceptions of telemedicine as a function of psychiatric diagnosis could not be analyzed.

During the study period, patients attended twice as many telemedicine (mean per patient, 3.6) than in-person visits (mean per patient, 1.9). This shows that, although patients still traveled to the clinic for face-to-face appointments periodically, they preferred telemedicine visits, when possible. Although not ideal for patients with depression or anxiety who tend to be socially isolated, telepsychiatry facilitates care for patients who would otherwise not find the energy or desire to travel to appointments. Patients saved considerable time and effort and, since many patients with movement disorders can no longer drive, telepsychiatry also allowed more flexibility for family members’ schedules. As such, this modality may help reduce caregiver burden. While internet-based interventions designed for caregivers have been effective in reducing caregiver burden and improving caregiver mood, self-efficacy, and social support [26, 27], further research needs to assess the impact of patient telepsychiatry visits on caregivers.

From the provider’s point of view, telepsychiatry provided several benefits. Barring technical difficulties, visits started and ended on time, allowing better time management. In a busy interdisciplinary practice, patients may be delayed due to traffic or because their same-day visits with other team members lasted longer than anticipated. Telepsychiatry closely mirrors home visits, allowing a rare view into patients’ living circumstances. Moreover, telepsychiatry visits facilitated a more comprehensive diagnostic impression: for example, a patient who had recently relocated had unpacked his books, indicating his mood improvement. Similar to the patients’ observations, the main disadvantages noted by providers were technical difficulties.

The present study had several limitations. The sample size was small and the group was heterogeneous, including patients with Alzheimer’s disease, chronic pain, and multiple sclerosis along with individuals with movement disorders. The initial survey was not previously validated. Parts of this survey (i.e., advantages/disadvantages and perceptions of telemedicine as compared to in-person visits) could have been repeated after completion of the study to evaluate changes in patient and/or caregiver attitudes. Initial perceptions of telemedicine were positive; this could have also influenced patient experience during visits, as reflected in high satisfaction scores. None of the participants who thought telemedicine would be better than in-person visits on initial surveys had any prior experience with it, which indicates high expectations to start with, perhaps idealizing this model. Also, all patients were already receiving psychiatric services at MDNC (telemedicine was only used for follow-up visits). A robust therapeutic alliance may have raised satisfaction levels, if patients sought the opportunity to provide positive feedback on their overall psychiatric care. It should also be noted that one third of patients did not complete surveys, and there were several significant differences between completers and non-completers. On average, non-completers were slightly younger and had a lower education level; this could be due to the fact that one patient in this group was 22 years old and had only graduated high school. Survey completers received three times more telemedicine visits than non-completers (mean number per patient, 4.6 vs. 1.5) and twice more total visits (mean number per patient, 6.6 vs. 3.1); as such, they may have been more motivated to respond. On the other hand, only two patients received the surveys and did not reply; the other nine did not receive the surveys. Standardized measures such as the Patient Health Questionnaire-9 (PHQ-9) [28] and Generalized Anxiety Disorder-7-item (GAD-7) [29] were not consistently collected; thus, clinical progress could not be evaluated. In future studies, the impact on patient care (i.e., clinical improvement, less frequent emergency room visits or calls to the practice) should be a primary outcome. Additionally, patients who participate in telepsychiatry visits should be compared to patients matched by age, sex, education, and neurological and psychiatric diagnosis who received in-person treatment. This comparison would allow researchers to draw conclusions on the benefits and limitations of telemedicine as compared to psychiatric treatment as-usual.

## Conclusions

Telepsychiatry is a feasible option for patients with movement disorders and psychiatric comorbidities, even in a busy interdisciplinary clinic. Patients and caregivers expressed high satisfaction with telepsychiatry visits, with lower ratings for technical aspects. Access to care was improved. Whenever available, telepsychiatry visits should be considered in addition to in-person treatment. Future studies are needed to explore other aspects such as cost effectiveness and impact on patient care outcomes and caregiver burden.

## Data Availability

The datasets generated and analyzed during the current study are not publicly available due participant confidentiality reasons but are available from the corresponding author on reasonable request.

## Abbreviations

CBT: Cognitive-behavioral therapy
DBS: Deep brain stimulation
GAD-7: Generalized Anxiety Disorder-7-item
MDNC: Movement Disorders and Neuromodulation Center
PD: Parkinson’s disease
PHQ-9: Patient Health Questionnaire-9
RACE-PD: Remote Access to Care, Everywhere, for Parkinson Disease
REDCap: Research Electronic Data Capture
SD: Standard deviation
UCSF: University of California, San Francisco
US: United States
