# Publisher Correction to: Journal of Clinical Movement Disorders, volume 6

**DOI:** 10.1186/s40734-019-0079-9

**Published:** 2019-08-07

**Authors:** Journal of Clinical Movement Disorders

**Affiliations:** London, UK

**Publisher Correction to: J Clin Mov Disord (2019) 6**

**https://doi.org/10.1186/s40734-019-0077-y**

An error occurred during the publication of an article in Journal of Clinical Movement Disorders. This article was published in volume 6 with a duplicate citation number.

In this correction article the old and new citation metadata are published in Table 1.

**Table 1:** Table 1

DOI	Incorrect citation number	Correct citation number
10.1186/s40734-019-0077-y	1	2

The original article has been updated. The publisher apologizes for the inconvenience caused to our authors and readers.

